# Thermophilic fermentation of acetoin and 2,3-butanediol by a novel *Geobacillus* strain

**DOI:** 10.1186/1754-6834-5-88

**Published:** 2012-12-06

**Authors:** Zijun Xiao, Xiangming Wang, Yunling Huang, Fangfang Huo, Xiankun Zhu, Lijun Xi, Jian R Lu

**Affiliations:** 1 State Key Laboratory of Heavy Oil Processing and Centre for Bioengineering & Biotechnology, China University of Petroleum, Qingdao, 266580, PR China; 2 Biological Physics Laboratory, School of Physics and Astronomy, University of Manchester, Manchester, M13 9PL, UK

**Keywords:** Acetoin, 2,3-butanediol, Thermophilic fermentation, *Geobacillus*, Novel metabolite, Key enzymes

## Abstract

**Background:**

Acetoin and 2,3-butanediol are two important biorefinery platform chemicals. They are currently fermented below 40°C using mesophilic strains, but the processes often suffer from bacterial contamination.

**Results:**

This work reports the isolation and identification of a novel aerobic *Geobacillus* strain XT15 capable of producing both of these chemicals under elevated temperatures, thus reducing the risk of bacterial contamination. The optimum growth temperature was found to be between 45 and 55°C and the medium initial pH to be 8.0. In addition to glucose, galactose, mannitol, arabionose, and xylose were all acceptable substrates, enabling the potential use of cellulosic biomass as the feedstock. XT15 preferred organic nitrogen sources including corn steep liquor powder, a cheap by-product from corn wet-milling. At 55°C, 7.7 g/L of acetoin and 14.5 g/L of 2,3-butanediol could be obtained using corn steep liquor powder as a nitrogen source. Thirteen volatile products from the cultivation broth of XT15 were identified by gas chromatography–mass spectrometry. Acetoin, 2,3-butanediol, and their derivatives including a novel metabolite 2,3-dihydroxy-3-methylheptan-4-one, accounted for a total of about 96% of all the volatile products. In contrast, organic acids and other products were minor by-products. α-Acetolactate decarboxylase and acetoin:2,6-dichlorophenolindophenol oxidoreductase in XT15, the two key enzymes in acetoin metabolic pathway, were found to be both moderately thermophilic with the identical optimum temperature of 45°C.

**Conclusions:**

*Geobacillus* sp. XT15 is the first naturally occurring thermophile excreting acetoin and/or 2,3-butanediol. This work has demonstrated the attractive prospect of developing it as an industrial strain in the thermophilic fermentation of acetoin and 2,3-butanediol with improved anti-contamination performance. The novel metabolites and enzymes identified in XT15 also indicated its strong promise as a precious biological resource. Thermophilic fermentation also offers great prospect for improving its yields and efficiencies. This remains a core aim for future work.

## Background

Acetoin (3-hydroxybutan-2-one) is widely used in food, flavour, cosmetics, and chemical synthesis [1]. Its reduced form, 2,3-butanediol, is not just limited to the manufacture of butadiene, or to its use as an antifreeze agent and a precursor of many synthetic materials including polymers and resins [2]. With a heating value of 27,200 J/g, 2,3-butanediol compares favourably with ethanol (29,100 J/g) and methanol (22,100 J/g) for use as a liquid fuel or fuel additive. Dehydration of 2,3-butanediol yields the industrial solvent methyl ethyl ketone, which can be hydrogenated to yield high octane isomers, suitable for high quality aviation fuels.

Acetoin and 2,3-butanediol can be produced by bacterial fermentation using renewable biomass. Such platform chemicals are regarded as potential alternatives of non-renewable fossil materials [3]. Both acetoin and 2,3-butanediol are metabolites of the acetoin metabolic pathway in bacteria. They can be transformed to each other by 2,3-butanediol dehydrogenase in cells. Acetoin and 2,3-butanediol sometimes coexist in fermentation broth. Because these two compounds are quite different in boiling points, they can be easily separated from each other by distillation.

The scientific understanding and the technological development leading to the production of acetoin and 2,3-butanediol have undergone four periods, evident from the number of the main publications. During the first period (1920s-1981), research work focused on product determination, the basic characterization of microbial properties and development of fermentation processes. Period II (1982–2001) addressed the main biological questions concerning pathways, enzymes, genes, and regulations [4]. This active phase was then followed by a few quiet years (Period III). Resurging from 2008, the current period features three words: cost-effective [5-7], efficient-recovery [8-10], genomics-guided [3,11]. But in general, fermentation study is one of the main themes throughout the entire development process.

To date, however, almost all these studies have only reported the production of acetoin and 2,3-butanediol from mesophilic fermentation processes or enzymatic reactions, typically at the temperature range of 30-40°C (for recent reviews, see [2,12]). To avoid bacterial contamination during these biotransformation processes, culture media must be strictly sterilized at high temperature and pressure, requiring the installation of specialized sterilization facilities and provision of steam and cooling water [13]. Operation of the sterilization facilities requires trained manpower and consumes energy. To worsen the situation, long time sterilization under high temperature causes serious nutrient loss and generates harmful chemicals that may in turn undermine normal fermentation.

In order to resolve these problems, thermophilic fermentation processes, typically operated at 50 – 60°C, have attracted attention over the past few years [13]. Recently Wang et al. reported the first high temperature fermentation of 2,3-butanediol using a genetically engineered bacterial strain, showing its great potential [14]. Because normal environmental microorganisms cannot reproduce above 45°C and vegetative cells are killed at higher temperature by pasteurization, thermophilic fermentation can thus reduce the risk of bacterial contamination. It is in fact possible to operate thermophilic fermentation even without sterilization [15], making it more efficient and cost-effective. In addition, thermophilic fermentation matches the simultaneous saccharification process which has the best efficiency around 55°C [14].

In this work, we report the isolation and characterization of a novel thermophilic bacterium for the production of acetoin and 2,3-butanediol and the related investigation of its novel metabolites and enzymes, aiming at developing a more efficient and effective fermentation process utilizing the thermophilic strain.

## Results

### Morphology, physiological characterization, phylogenetic analysis, and growth on crude oil

Produced water (PW) is the water that is produced along with the crude oil. This isolate XT15 was screened from a PW-based glucose-peptone-yeast extract (PW-GPY) culture medium after 100°C sterilization. The strain was found to grow well on GPY agar, yielding large (about 4 mm in diameter, 2 d), white (to pale pink for old colonies), irregular edged, surface-wrinkled, and opaque colonies. Scanning electron microscopy (SEM) micrographs of XT15 revealed rod-shaped cells of 0.4-0.5 μm × 0.9-1.8 μm in size. Some of the physiological characteristics of XT15 are given in Table 1.

**Table 1:** Physiological characteristics of strain XT15

**Characteristics**		**Result**
Gram staining		+
Voges-Proskauer test		+
Hydrogen sulfide production		-
Gelatin hydrolysis		+
Enzyme production	Urease	-
	Ornithine decarboxylase	-
	Lysine dihydrolase	-
	Arginine dihydrolase	-
Carbohydrates tests	Glucose	+
	Sucrose	+
	Arabinose	+
	Inositol	-
	Lactose	-
	Rhamnose	-
	Amygdalin	+
	Melibiose	-

+, positive reaction; −, negative reaction.

**Figure 1:**
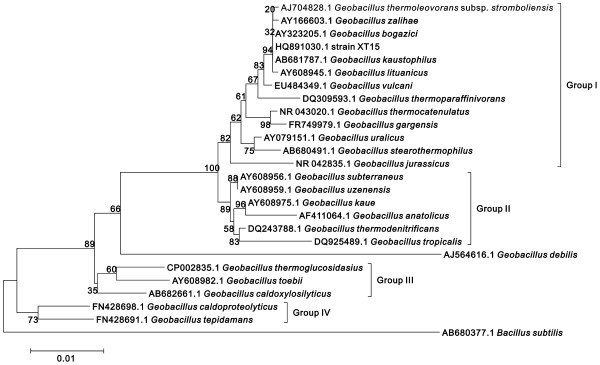
**Phylogenetic relationship between strain XT15 and *Geobacillus *species (with GenBank accession numbers) based on 16S rRNA sequences.** The tree was constructed with the MEGA5 program using the neighbour-joining cluster algorithm. The numbers at the branches are bootstrap values (confidence limits). *Bacillus subtilis* was used as an outgroup to root the tree. Scale bar, 0.01 substitutions/site.

There are four main branches or groups in the phylogenetic tree of the genus *Geobacillus* derived from the similarity of the 16S rRNA sequences of *Geobacillus* species (Figure 1). Group I is the largest branch containing some common *Geobacillus* species such as *G. stearothermophilus* (the type species of the genus *Geobacillus*), *G. thermoleovorans*, and *G. kaustophilus*. Strain XT15 (GenBank accession No. HQ891030.1) is clearly a member of Group I and it is closest to *G. kaustophilus* and *G. bogazici*. This strain has been designated as *Geobacillus* sp. XT15 and deposited in China Center for Type Culture Collection with deposition number M2011022.

**Figure 2:**
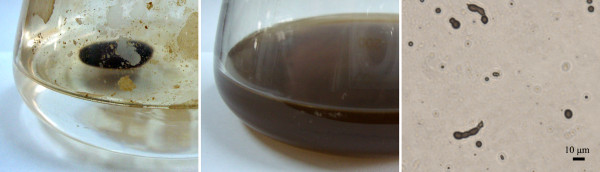
**Growth of XT15 in a crude oil-based mineral-salts medium at 55°C.** Left, the control experiment using heat-inactivated XT15 cells; middle, bacterial growth of XT15 using crude oil as the sole carbon source; right, observation of the culture medium in the middle through a light microscope.

Compared with other thermophilic bacteria, the ability of degradation and utilization of hydrocarbons at high temperature is another important feature of *Geobacillus* species [16]. After one week of aerobic cultivation of XT15 at 55°C in a crude oil-based mineral-salts (MS) medium, obvious bacterial growth was observed. The crude oil floating on the liquid surface disappeared and the colour of the medium changed into brown (Figure 2, middle), while no change took place in the control even under a prolonged period (Figure 2, left). Observation through a light microscope revealed that the large drops of crude oil were dispersed into micron-sized droplets (Figure 2, right), indicating the production of biosurfactants (as emulsifier) by XT15.

### Environmental growth conditions: growth temperature, pH and dissolved oxygen (DO)

**Figure 3:**
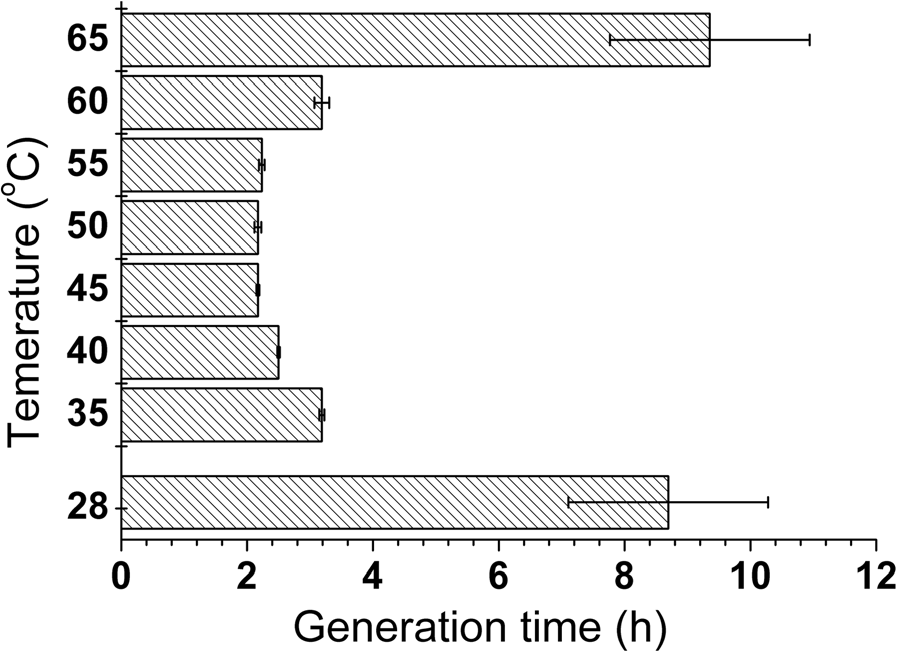
**Generation time of *Geobacillus *sp. XT15 under different temperature conditions.**

For temperature optimization, experiments were performed at 28°C, 35°C, 40°C, 45°C, 50°C, 55°C, 60°C, 65°C, and 70°C, respectively. Generation time (doubling time) was calculated according to the specific growth rate. As shown in Figure 3, XT15 gained the shortest generation time between 45°C and 55°C and it would double its biomass every 2 h in the GPY medium. Therefore, XT15 was examined as an obligate thermophile, although there was no visible growth at 70°C during a one-week period cultivation.

**Figure 4:**
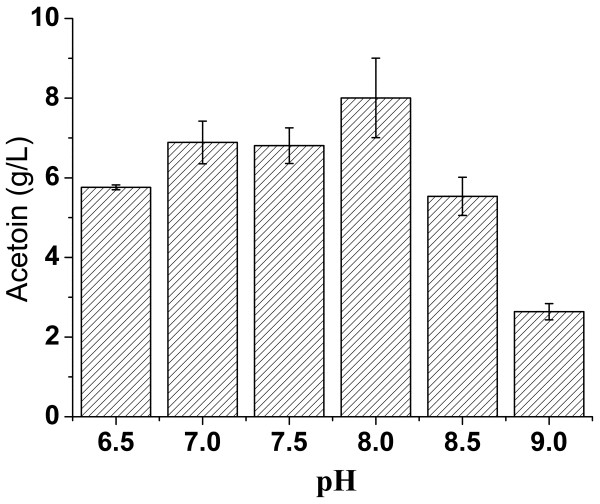
**Acetoin yield of *Geobacillus *sp. XT15 using GPY media of different initial pH values.**

For pH optimization, the pH values of the fermentation media were adjusted to 6.5, 7.0, 7.5, 8.0, 8.5, and 9.0, respectively, before autoclaving. As shown in Figure 4, the best yield of acetoin was 8.0 g/L when the initial pH of the culture medium was set at 8.0. Glucose (20 g/L) was completely depleted and no 2,3-butanediol was detected in the final fermentation broth. The product yield (0.40 g/g) was at 82% of the theoretical value (0.49 g/g). A trace amount of lactic acid (less than 1 g/L) was detected as well in the fermentation broth.

The effect of DO on bacterial growth and product yield was tested by either changing the medium loading volume in shaking flasks or comparing aerobic and anaerobic cultivation. Loading 30 or 20 mL medium per flask did not alter the yield of acetoin. This was because when DO was above a certain level (the critical DO concentration), changes in DO concentration would not affect production any longer. However, under anaerobic condition, no growth or product was detected during a one-week period cultivation of XT15.

### Effects of culture medium: carbon source and nitrogen source

**Figure 5:**
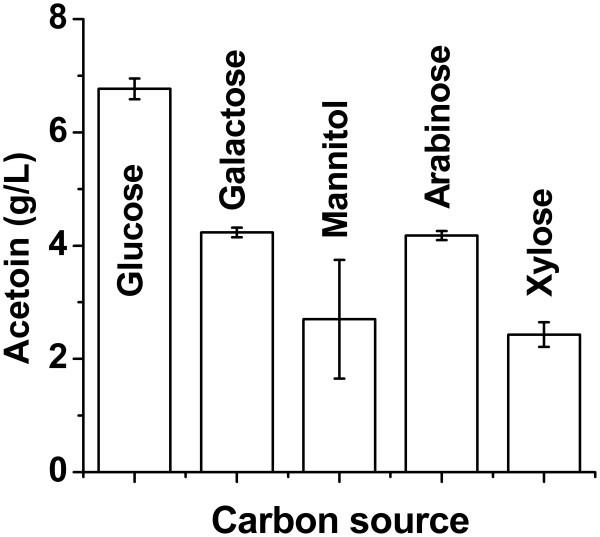
The comparison of acetoin production using different carbon sources.

Galactose, mannitol, arabionose, and xylose were used as carbon source individually and their influences were compared with glucose (control). As shown in Figure 5, about 1/3 ~ 2/3 of the yield of glucose was achieved using the substitutes. As these carbon substrates are the hydrolysis products of biomass, *Geobacillus* sp. XT15 could be further developed to utilize cellulosic biomass as the feedstock.

**Figure 6:**
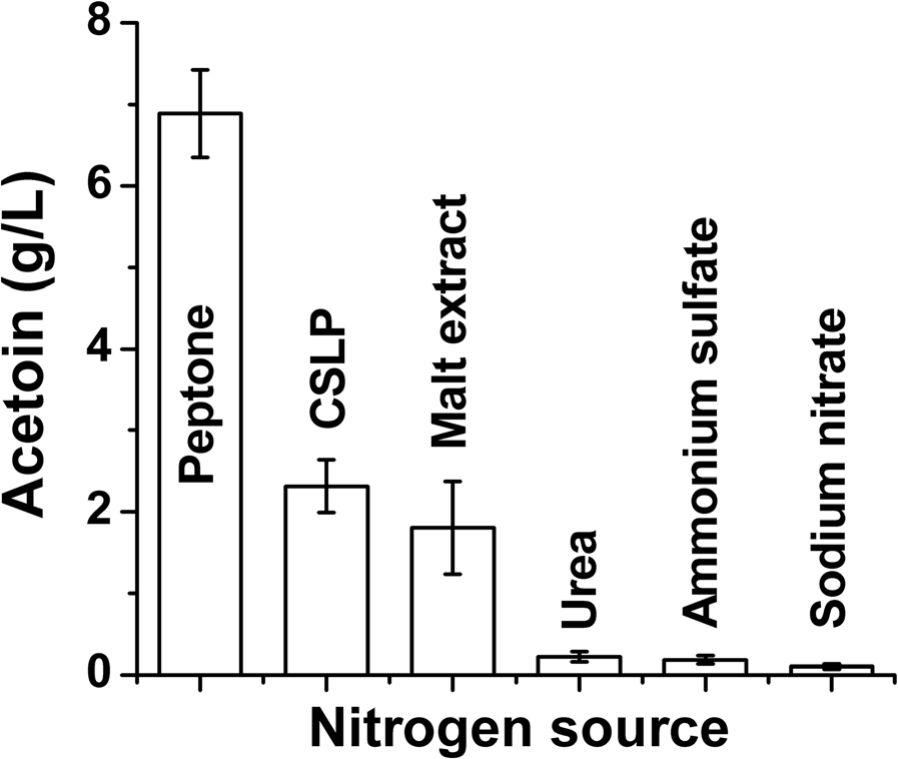
The comparison of acetoin production using different nitrogen sources.

For nitrogen source screening, peptone, spray-dried corn steep liquor powder (CSLP), malt extract powder, urea, ammonium sulfate, and sodium nitrate were tested (Figure 6). Peptone showed the best performance, followed by CSLP and the malt extract powder. Urea and inorganic nitrogen resulted in very poor production (no more than 0.5 g/L). However, the price of peptone is too high for bulk fermentative products including acetoin and 2,3-butanediol. Among all the nitrogen sources studied, CSLP was most cost-effective and the influence of CSLP concentration on acetoin and 2,3-butanediol yield was further tested.

**Figure 7:**
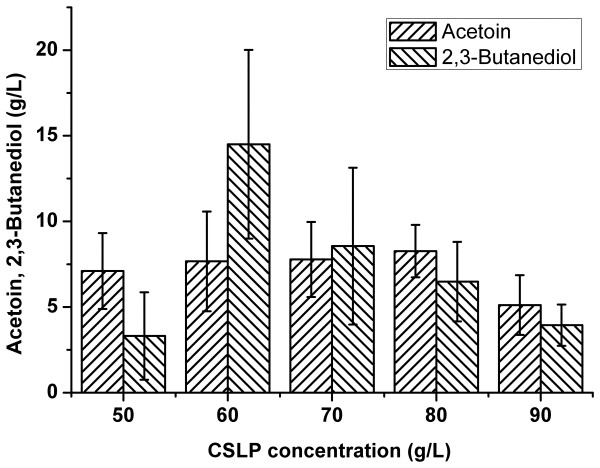
Acetoin and 2,3-butanediol production using variable amounts of CSLP as the nitrogen source.

As shown above, glucose was a suitable carbon substrate. The original concentration of glucose in the GPY medium formulation (i.e. 20 g/L) was far from enough to support the maximum acetoin and 2,3-butanediol production. Then glucose concentration in the medium was added up to 220 g/L. Bacterial growth was not influenced by possible substrate inhibition and the high osmotic pressure (data not shown). Therefore, the fermentation medium was changed to: glucose at 220 g/L, CSLP from 50 to 90 g/L, yeast extract at 10 g/L, deionized water, pH 8.0. As shown in Figure 7, acetoin and 2,3-butanediol gained the highest yields (7.7 g/L of acetoin and 14.5 g/L of 2,3-butanediol) after 2 days of fermentation at the CSLP concentration of 60 g/L. The concentration of the residual glucose in the fermentation broth was 155 g/L. The product yield was at 69% of the theoretical value, lower than the above mentioned yield of 82%.

### Volatile products in the cultivation broth of XT15

**Figure 8:**
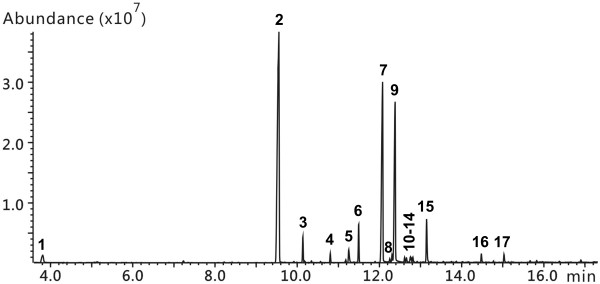
TIC of the GC-MS analysis to show the occurrence of the 17 peaks in the extract of the fermentation broth.

During the organic solvent extraction process, the addition of a large amount of salt into the cultivation broth was helpful to improve the extraction efficiency. Low pH was effective for the extraction of organic acids. By gas chromatography (GC) analysis, no additional substances (GC peaks) appeared when the aqueous phase was changed from acidic to neutral or alkaline pH. As shown in Figure 8, there are 17 main GC peaks in the extract of the cultivation broth of XT15.

Peaks 1, 2, 7, and 9 could be easily recognized as diacetyl, acetoin, 2,3-butanediol, and 2,3-butanediol, respectively, in gas chromatography–mass spectrometry (GC-MS) identification. There are 3 stereoisomers of 2,3-butanediol: *(2R, 3R)*-, *(2S, 3S)*-, and *meso*-2,3-butanediol. The first two kinds are racemic mixtures and they can only be separated from each other in a chiral column [1]. But the racemic mixtures (or just one of the two racemic stereoisomers) can be easily separated from the *meso* form by ordinary non-chiral GC capillary columns.

Ammonia was generated in the medium by the oxidative deamination of amino acids. Nitrogen-containing heterocyclic compounds could be formed from acetoin and ammonia by non-biological processes [17]. Control experiments without inoculum (but adding 10 g/L of acetoin and 10 g/L of diammonium phosphate in the medium) were performed. Identical peaks of 2,3,5,6-tetramethylpyrazine (TTMP) and an oxazoline (1-(2,4,5-trimethyl-2,5-dihydrooxazol-2-yl)ethanol, OXZ) were detected as Shu and Lawrence reported [17] in both experiments (with or without inoculum). OXZ produced multiple peaks because it has 3 chiral carbon atoms in its molecule. Therefore, peak 6 and peaks 10–13 in Figure 8 were identified as TTMP and OXZ, respectively. Peak 4 was identified as 2,3,5-trimethylpyrazine (TMP), a common by-product of TTMP [18]. As mentioned by Larroche et al. [19], 1-aminopropan-2-one could be generated from L-threonine. Then 1-aminopropan-2-one, acetoin, and ammonia would synthesize TMP spontaneously in aqueous solution.

**Figure 9:**
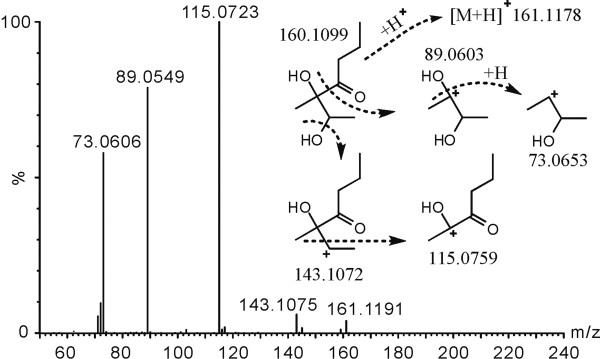
**Proposed CI MS fragmentation pathway of the novel product butyrylbutanediol.** Numbers beside the chemical structures are their theoretical values of m/z.

Acetylbutanediol (3,4-dihydroxy-3-methylpentan-2-one), prepared as described before [20], was used as a standard substance to help check peaks. Peak 3 was identified as acetylbutanediol because they shared identical retention time and mass spectrum. The mass spectrum of peak 15 was similar to that of acetylbutanediol. By further identification with high-resolution GC-MS in CI (Chemical Ionization) mode, peak 15 was tentatively inferred as butyrylbutanediol (2,3-dihydroxy-3-methylheptan-4-one) from mass spectral fragmentation (Figure 9).

In addition, 4 organic acids and phenylethyl alcohol were determined in the products of XT15. Therefore, all the main volatile products were identified as shown in Table 2. According to the integral area of the peaks in the total ion chromatogram (TIC) (Figure 8), acetoin, 2,3-butanediol, and their derivatives accounted for a total of about 96%. Organic acids and other products accounted for about 4%.

**Table 2:** Identification results of volatile products

**#**	**RT**	**Ratio**	**Common name or abbr.**	**IUPAC systematic name**
1	3.810	1.39	diacetyl	butane-2,3-dione
2	9.554	42.53	acetoin	3-hydroxybutan-2-one
3	10.139	1.79	acetylbutanediol	3,4-dihydroxy-3-methylpentan-2-one
4	10.807	0.67	TMP	2,3,5-trimethylpyrazine
5	11.255	1.29	acetic acid	acetic acid
6	11.498	2.72	TTMP	2,3,5,6-tetramethylpyrazine
7	12.076	23.21	2,3-butanediol	butane-2,3-diol
8	12.310	0.57	isobutyric acid	2-methylpropanoic acid
9	12.381	18.95	2,3-butanediol	butane-2,3-diol
10	12.615	0.35	OXZ	1-(2,4,5-trimethyl-2,5-dihydrooxazol-2-yl)ethanol
11	12.661	0.39	OXZ	1-(2,4,5-trimethyl-2,5-dihydrooxazol-2-yl)ethanol
12	12.752	0.35	OXZ	1-(2,4,5-trimethyl-2,5-dihydrooxazol-2-yl)ethanol
13	12.772	0.34	OXZ	1-(2,4,5-trimethyl-2,5-dihydrooxazol-2-yl)ethanol
14	12.814	0.48	butanoic acid	butanoic acid
15	13.149	3.68	butyrylbutanediol	2,3-dihydroxy-3-methylheptan-4-one
16	14.483	0.65	hexanoic acid	hexanoic acid
17	15.032	0.65	phenylethyl alcohol	2-phenylethanol

The # symbols are in accordance with peaks in Figure 8. RT, retention time in min. Ratios in percentages were calculated from integral areas of all the main peaks in the TIC using the normalization method.

### Effects of temperature on enzyme activities

**Figure 10:**
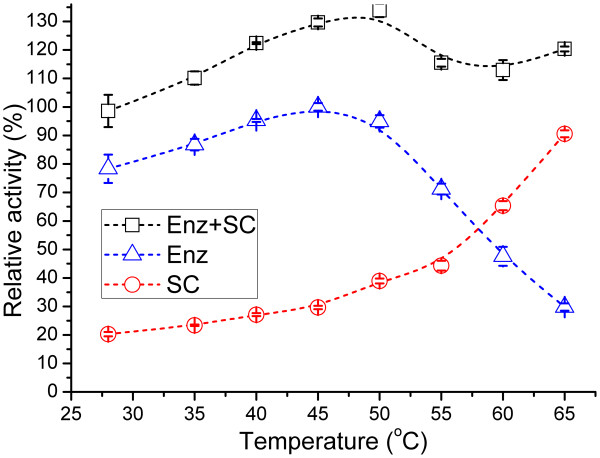
**Effects of temperature on ALDC activity in *Geobacillus *sp. XT15.** Enz, enzyme; SC, spontaneous cleavage. The relative activity of each temperature point was calculated by comparing with the maximum enzyme activity at 45°C, which was set to 100%.

**Figure 11:**
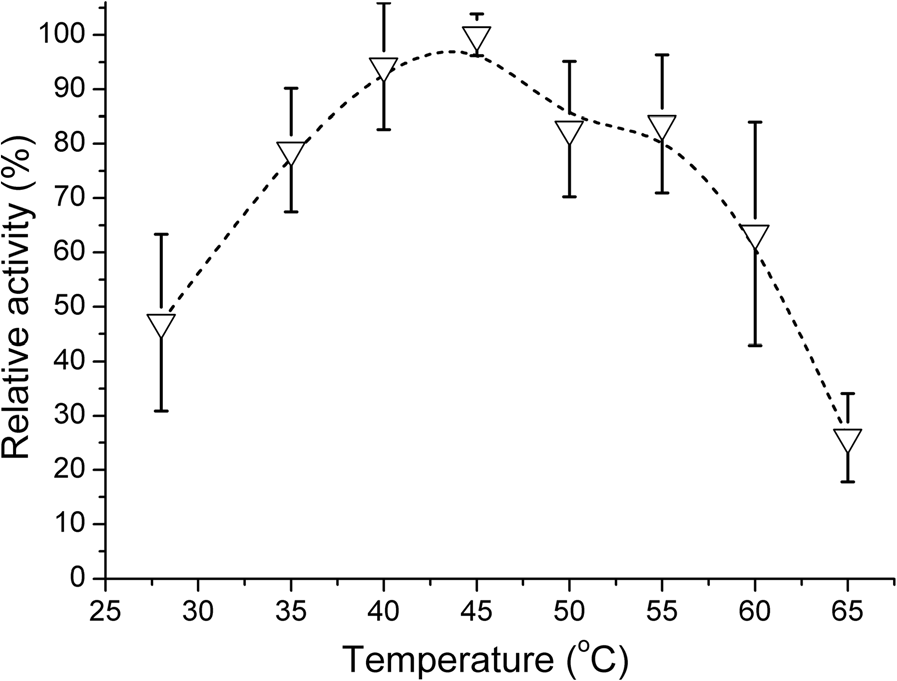
**Effects of temperature on AoDH ES E1 activity in *Geobacillus *sp. XT15.** The relative activity of each temperature point was calculated by comparing with the maximum enzyme activity at 45°C, which was set to 100%.

α-Acetolactate decarboxylase (ALDC) and acetoin:2,6-dichlorophenolindophenol oxidoreductase (Ao:DCPIP OR or AoDH ES E1) are the key enzymes in acetoin biosynthesis pathway and catabolism pathway, respectively. As shown in Figures 10 and 11, the two enzymes shared the same optimum temperature of 45°C and both of them had a wider active temperature range (80% activity from 28 to 53°C for ALDC and from 35 to 55°C for E1, respectively). Furthermore, both enzymes retained 50% activity at around 60°C. Although the two profiles seem different, both enzymes could be considered to be moderately thermophilic.

The spontaneous cleavage (SC) of α-acetolactate should not be neglected as shown in Figure 10, especially when the temperature was above 50°C. When the activity of SC was taken into account, α-acetolactate could be transformed into acetoin with maximum efficiency at 50°C. Therefore, the total cleavage activity of α-acetolactate remained high in the temperature range from 28 to 65°C in *Geobacillus* sp. XT15.

## Discussion

By isolation from different environments [21] or genetic manipulations [14,22,23], dozens of species from various genera, such as *Bacillus*[24,25], *Klebsiella*[26], *Enterobacter*[27], *Serratia*[28], and *Paenibacillus*[29,30] have been obtained for potential industrial production of acetoin and/or 2,3-butanediol. However, none of these strains is a naturally occurring thermophile. To our best knowledge, XT15 is the first naturally occurring thermophilic acetoin and/or 2,3-butanediol producer. It is thus of great value to both practical application and theoretical study.

During enhanced oil recovery process, additional water is often injected into reservoirs to help force the crude oil to the surface. Both the injected water and the natural water lying under the hydrocarbons are eventually produced along with the oil. Therefore, the bacteria collected from PW may come from ground or underground. In recent years, *Bacillus* strains, which were known as acetion producers, were also isolated from the PW of Shengli Oilfield, China. *Geobacillus* species were regarded as a branch of the *Bacillus* genus before Nazina et al. reclassified them in 2001 [16]. Strain XT15 shared most physiological characteristics with common *Geobacillus* species, including the ability of hydrocarbon utilization. But XT15 is also capable of acetoin excretion (positive in Voges-Proskauer test), which is unusual in the genus *Geobacillus*[16]. The record product yield achieved was at 82% of the theoretical value. In other words, 82% of glucose was converted into acetoin, indicating that the acetoin metabolic pathway is a dominant pathway in *Geobacillus* sp. XT15. This would distinguish XT15 from other *Geobacillus* species. The substrate conversion rate of XT15 was equivalent to that of *Bacillus subtilis* CICC 10025 [24].

According to Nazina et al. [16], *Geobacillus* species are chemoorganotrophic, obligately thermophilic, aerobic or facultatively anaerobic, Gram positive, rod-shaped bacteria. Most species are widely distributed in nature. Oxygen is the electron acceptor, replaceable in some species by nitrate. The growth-temperature range is 37-75°C, with an optimum at 55-65°C. Growth occurs in a pH range of 6.0 to 8.5, with an optimum at pH 6.2-7.5. Therefore, the optimum temperature of XT15 is relatively lower, but the pH range is a bit higher.

Optimization of culture medium components, especially carbon source and nitrogen source, is not only yield-relevant but also cost-effective. Acetoin and 2,3-butanediol are important primary metabolites like ethanol, acetate, and lactate in various microorganisms and the excretion of them has vital physiological implications [4]. Since production of acetoin and 2,3-butanediol is generally accepted to be a growth-associated phenomenon, the conditions for the maximum product formation must approximately be the same as those for the maximum biomass yield [31].

The influences of fermentation ages on acetoin and 2,3-butanediol production were also studied (unpublished data). The maximum yield could usually be achieved 1–3 h after the time point when the carbon source was depleted in the culture medium. But if the carbon source was excessive and could not be used up, the yield usually remained relatively stable after it grew to a certain extent. However, these finding were not always repeatable. The quality of seed culture had great influence on the maturation age of a fermentation process. But generally the time point of the maximum product yield was approximately the same as that for the maximum biomass yield. In Figures 4, 5, and 6, when 20 g/L of glucose was used as the carbon source, glucose could usually be depleted in 8 h and the maximum product yield and biomass yield could be achieved at about 10 h. In Figure 7 when 220 g/L of glucose was used, maximum yields could be achieved at about 48 h. All these findings again supported the notion that bacterial production of acetoin and 2,3-butanediol is a growth-associated phenomenon.

A noticeable observation is that the ratios between acetoin and 2,3-butanediol in the cultivation broth were variable. This could be explained by the physiological state of the bacteria, which would be substantially influenced by the culture medium, especially the content of the carbon source. When the concentration of glucose was low (as shown in Figures 4, 5, 6), the product would be acetoin. As the concentration of glucose was elevated to a certain higher level, 2,3-butanediol would arise as another important product. This phenomenon could be linked to the cellular NADH/NAD levels. The reversible transformation between acetoin and 2,3-butanediol couples with the NADH/NAD conversion, participating in the regulation of the cellular NADH/NAD levels [32,33]. Glucose not only provides energy and anabolic materials but also sustains a reducing cellular environment, protecting cells from oxidative stress. When glucose was abundant and the system was maintained under a highly reducing environment, XT15 cells yielded 2,3-butanediol to store the reduced force. As glucose was kept at a low level or depleted, the product would be acetoin and even the accumulated 2,3-butanediol would be oxidized into acetoin to generate NADH and maintain NADH/NAD homeostasis [4].

Including metabolites and non-enzymatic products, a total of 14 substances (lactic acid and 13 volatile products) were identified from the fermentation broth of *Geobacillus* sp. XT15 when this strain was cultivated in media containing glucose, peptone and yeast extract. TMP, TTMP, and OXZ were believed to be non-enzymatic products. As for metabolites, XT15 shares great similarity with certain *Bacillus* species. In addition to acetoin, 2,3-butanediol, diacetyl and acetylbutanediol, some *Bacillus* strains can produce mixtures of lactic, isovaleric, isobutyric and acetic acids [34]. The trace amount of phenylethyl alcohol detected in the cultivation broth would be the product of L-phenylalanine, as in certain microorganisms [35,36]. As for the novel product butyrylbutanediol, the interpretation of the formation process could be inspired by the formation of acetybutanediol [20]. Butyryl-CoA is readily generated from butanoic acid by CoA-transferase or hexanoic acid by the beta-oxidation pathway. Then it is postulated that butyryl-CoA and acetoin produced butyrylbutanediol by AoDH ES E1, which was also detected in this study.

The first step of the acetoin biosynthesis pathway is the reaction from pyruvate to α-acetolactate catalysed by two enzymes: the catabolic α-acetolactate-forming enzyme and the anabolic α-acetolactate-forming enzyme [4]. However, there is only one enzyme catalysing the transformation from α-acetolactate to acetoin, i.e. ALDC. Therefore, ALDC was regarded as the key enzyme and the marker of the acetoin biosynthesis pathway. Compared to the published work [37,38], the ALDC of XT15 obtained a higher optimum temperature and a wider active range at higher temperatures. When glucose was depleted in the medium, AoDH ES could be induced by acetoin and then acetoin would be reutilized as a substitutional carbon source. The initial step of acetoin breakdown was catalysed by AoDH ES E1 in the catabolic pathway of acetoin in bacteria [4]. Only AoDH ESs from mesophilic bacteria were characterized before Payne et al. [39] reported the AoDH ES from a hyperthermophilic archaeon *Sulfolobus solfataricus* with an optimum temperature of 80°C. This study thus provided an example of a moderately thermophilic AoDH ES. ALDC and AoDH ES E1 were both active in *Geobacillus* sp. XT15, indicating that the acetoin metabolic pathway was active in this strain. The optimum temperatures and active temperature ranges of the two key enzymes were consistent with those of growth temperatures, respectively, indicating that the acetoin metabolic pathway originally existed in this bacterium. The acetoin metabolic genes in XT15 could have experienced similar evolutionary processes or even affected with each other with *Bacillus* by horizontal gene transfer, in consideration of the great similarities between *Geobacillus* sp. XT15 and some *Bacillus* species.

## Conclusions

A novel thermophilic acetoin and 2,3-butanediol producer XT15 was isolated. It is an aerobic, gram positive, rod-shaped *Geobacillus* bacterium. At its optimum growth temperature range of 45-55°C, XT15 cells would reproduce every 2 h. The optimum initial pH of the culture medium is 8.0. XT15 prefers organic nitrogen sources like CSLP and has the potential to utilize cellulose hydrolysates. 14 products were identified in its cultivation broth, including a novel metabolite. Its two key enzymes in acetoin biosynthesis pathway and catabolism pathway shared identical optimum temperature of 45°C and wide active temperature range. As a novel naturally occurring thermophilic strain for the production of acetoin and/or 2,3-butanediol, there is potential to enhance its yields in further studies.

## Methods

### Chemicals and reagents

2,6-Dichloroindophenol sodium salt hydrate (≥ 97.0%), ethyl 2-acetoxy-2-methylacetoacetate (97%), and thiamine pyrophosphate (≥ 95%) were bought from Sigma-Aldrich. Natural acetoin (GC ≥ 96%), a fermentative product from glucose, was obtained from Shanghai Apple Flavor & Fragrance Co., Ltd. (China). 2,3-Butanediol (GC ≥ 97%), a mixture of racemic and meso forms, was bought from Sinopharm Chemical Reagent Co., Ltd. (China). Other chemicals used in this study were all commonly available analytical reagents. Spray-dried CSLP used in this study was an industrial grade product from Shandong Zouping Hebanshan Tianyuan Chemical Co., Ltd. (China). The composition of CSLP is shown in Table 3.

**Table 3:** Composition of CSLP used in this study

**Test items**	**Concentration**	**Test items**	**Concentration**
Protein	43.58%	Lactic acid	0.39%
Fat	0.80%	Water	5.61%
Total sugar	3.14%	Alanine	1.69%
Ash	16.72%	Arginine	0.95%
Sulfate	4.33%	Aspartate	0.41%
Sulfite	2.66%	Cystine	0.04%
Reactive phosphorus	0.91%	Glutamate	0.22%
Total phosphorus	4.01%	Glycine	0.35%
Chloride	2.03%	Histidine	0.30%
Potassium	5.66%	Isoleucine	0.59%
Magnesium	1.50%	Leucine	2.25%
Calcium	0.41%	Lysine	0.47%
Iron	0.03%	Methionine	0.36%
Zinc	217.5 mg/kg	Phenylalanine	0.90%
VB1	19.04 mg/kg	Proline	1.34%
VB2	11.62 mg/kg	Serine	0.37%
VB6	44.22 mg/kg	Threonine	0.46%
Nicotinic acid	1798 mg/kg	Tyrosine	0.30%
Biotin	16.18 mg/kg	Valine	1.07%

### Microorganism screening

GPY (glucose 20 g/L, peptone 20 g/L, yeast extract 10 g/L, deionized water, pH 7.0) or modified GPY media were used in this study. Glucose was autoclaved separately. The oil well PW from Shengli Oilfield (Dongying, China) was used to replace deionized water for the preparation of screening culture medium (PW-GPY medium). The PW-GPY medium was loaded into Erlenmeyer flasks and heated to 100°C rapidly. Without delay the flasks were then transferred into a shaking bath controlled at 60°C. After 2 d enrichment, the culture was streaked onto GPY agar and incubated at 60°C for strain isolation. Colonies larger in diameters and with deeper red colour in Voges-Proskauer tests were picked up and inoculated into GPY medium for final screening.

### Isolate identification and observation of bacterial growth on crude oil

Physiological characterization was done using a bacterial biochemical identification kit (Qingdao Hope Bio-Technology Co., Ltd., China) according to the manufacturer’s instructions. The partial 16S rRNA gene sequence of the isolate was routinely amplified using universal primers 27 F and 1492R. The PCR products were purified and sequenced using an ABI 3730 DNA Analyzer (Applied Biosystems) by Shanghai Sangon (China). Searches of the GenBank database were conducted by using BLAST [40]. 16S rRNA sequences of representative homologous species were retrieved from GenBank to generate the phylogenetic tree (1,000 bootstrap replicates) using the maximum composite likelihood method and the neighbour-joining method in the MEGA5 program [41].

Bacterial growth of XT15 on crude oil was tested in a MS medium containing 500 mg/L of crude oil (from Shengli Oilfield, Dongying, China), 2.28 g/L of K_2_HPO_4_·3H_2_O, 0.47 g/L of NaH_2_PO_4_·2H_2_O, 1.32 g/L of (NH_4_)_2_SO_4_, 0.12 g/L of MgSO_4_, and 1 ml/L of trace element solution (2.63 g/L of CaCl_2_, 0.72 g/L of FeSO_4_·7H_2_O, 0.46 g/L of ZnSO_4_·7H_2_O, and 0.22 g/L of MnSO_4_·H_2_O). To avoid the entrainment of nutrients from inoculum, the cells in the seed culture were harvested by centrifugation and washed with sterile saline before inoculated into the MS medium. Control experiments were performed using heat-inactivated XT15 cells.

### Optimization of fermentation process and medium components

Cells from slant cultures were inoculated into a pH modified GPY medium (initial pH changed from 7.0 to 8.0) to prepare the seed culture. After 6–8 h incubation at 55°C with agitation, the grown seed culture was inoculated (5%, v/v) into appropriate media for optimization experiments including temperature, medium initial pH, DO, carbon source, and nitrogen source. Unless otherwise noted, all the optimization experiments were performed in triplicates using 100 mL Erlenmeyer flasks (loading 30 mL medium per flask) in a reciprocal shaking water bath controlled at 170 rpm and 55°C. For anaerobic cultivation tests, 200 mL serum bottles fully filled with medium were applied and stirrer bars were placed into the serum bottles to keep the bacterial cells well suspended.

### Analytical methods

Daily cell observation was performed using a light microscope (DMI3000B Microscope, Leica). Scanning electron microscopy analysis was performed routinely [42] using JSM-6390LV (JEOL). Bacterial growth was measured spectrophotometrically at 600 nm (OD_600_) and dry cell weight (DCW) was calculated from OD_600_ with a linear correlation factor (1 OD_600_ = 0.39 DCW g/L). The pH values of the cultivation broth were determined using pH 211 (HANNA instruments). Glucose and lactic acid concentrations were enzymatically determined using an SBA-40C biochemical analyser (Biology Institute of Shandong Academy of Sciences, China). Acetoin and 2,3-butanediol concentrations in the fermentation broth were analysed periodically with an Agilent 7890A GC equipped with a flame ionization detector and a 30-m HP-5 capillary column (HP Agilent). Prior to GC analysis, the samples were extracted using ethyl acetate with the addition of isoamyl alcohol as the internal standard. The operating conditions were as follows. Nitrogen was used as the carrier gas. The injector temperature and detector temperature were both set at 280°C. The column oven was maintained at 40°C for 2 min and then programmed to increase to 130°C at a rate of 20°C/min.

### Identification of volatile products

In GPY medium fewer kinds of volatile products were detected. In order to make more metabolites detectable, XT15 was cultured in an enhanced GPY medium with elevated carbon substrate and nitrogen substrate concentrations (glucose 220 g/L, peptone 80 g/L, yeast extract 10 g/L). After 48 h of cultivation at 170 rpm and 55°C, 150 mL of fermentation broth was centrifuged at 8000 *g* for 5 min and then 30 g of NaCl was added into the supernatant. Then the supernatant was adjusted to pH 2.0 using 6 M hydrochloric acid before extraction with 20 mL of dichloromethane. The organic phase was concentrated 10 times before GC-MS analysis using Agilent 7890A-5975C equipped with a 30 m HP-INNOWax Polyethylene Glyco column (0.25 mm inside diameter, 0.25 μm film thickness). Helium was used as the carrier gas. The column oven temperature was initially maintained at 40°C for 5 min and then ramped to 200°C at 15°C min^-1^, maintained at 200°C for 1 min and then ramped to 230°C at 20°C min^-1^. Mass spectra in the electron impact mode were generated at 70 eV and scan mode in the range of 20–400 amu. The compounds were tentatively identified by comparing their mass spectra with those contained in the MS data system (NIST08.L library). For the further identification of unknown products, high-resolution GC-MS in CI mode was also performed using Waters GCT Premier.

### Determination of enzyme activities

Strain XT15 was cultured in GPY medium (20 g/L of glucose) and samples were collected at different stages for the analysis of different enzymes. After culturing for 5 h (glucose was not depleted at this time), the cells were harvested by centrifugation for the determination of ALDC activity. About 5 h after glucose was depleted in the culture medium, the cells were harvested for the determination of AoDH ES E1. The harvested cells were resuspended in 50 mM phosphate buffered saline (pH 7.0) and disrupted by sonication. The disrupted cells were centrifuged at 10,000 *g* for 20 min at 4°C, and the supernatant was used as the crude cell extract. ALDC activity was assayed by measuring the amount of acetoin generated from α-acetolactate according to Stormer [43]. The creatine-naphthol colorimetric method was applied for acetoin measurement at 522 nm using a UV/visible spectrophotometer (DR 5000, HACH). The formation of acetoin in the enzymatic reactions was also confirmed by the GC method. AoDH ES E1 was assayed by measuring the reduction of DCPIP at 578 nm according to Steinbüchel et al. [44]. All the enzymatic experiments were performed in triplicates and the reactions without the crude extracts were set as blanks.

## Abbreviations

PW: Produced water; GPY: Glucose-peptone-yeast extract; SEM: Scanning electron microscopy; MS: Mineral-salts; DO: Dissolved oxygen; CSLP: Corn steep liquor powder; GC: Gas chromatography; GC-MS: Gas chromatography–mass spectrometry; TTMP: 2,3,5,6-tetramethylpyrazine; OXZ: 1-(2,4,5-trimethyl-2,5-dihydrooxazol-2-yl)ethanol; TMP: 2,3,5-trimethylpyrazine; CI: Chemical ionization; TIC: Total ion chromatogram; ALDC: α-acetolactate decarboxylase; Ao:DCPIP OR or AoDH ES E1: Acetoin:2,6-dichlorophenolindophenol oxidoreductase; SC: Spontaneous cleavage.

## Competing interests

The authors declare that they have no competing interests.

## Authors’ contributions

ZX designed the study, performed experiments, analysed the data and drafted the manuscript; XW and YH executed the experimental work and some data interpretation; FH, XZ, and LX participated in the experimental work; JRL coordinated the study and revised the manuscript. All authors read and approved the final manuscript.

## References

[B1] Xiao Z Lv C Gao C Qin J Ma C Liu Z Liu P Li L Xu P A novel whole-cell biocatalyst with NAD+ regeneration for production of chiral chemicals PLoS One 2010 5 e8860 10.1371/journal.pone.0008860 20126645 PMC2811184

[B2] Ji XJ Huang H Ouyang PK Microbial 2,3-butanediol production: a state-of-the-art review Biotechnol Adv 2011 29 351 364 10.1016/j.biotechadv.2011.01.007 21272631

[B3] Xu Y Wang A Tao F Su F Tang H Ma C Xu P Genome sequence of Enterobacter cloacae subsp. dissolvens SDM, an efficient biomass-utilizing producer of platform chemical 2,3-butanediol J Bacteriol 2012 194 897 898 10.1128/JB.06495-11 22275097 PMC3272950

[B4] Xiao Z Xu P Acetoin metabolism in bacteria Crit Rev Microbiol 2007 33 127 140 10.1080/10408410701364604 17558661

[B5] Cheng KK Liu Q Zhang JA Li JP Xu JM Wang GH Improved 2,3-butanediol production from corncob acid hydrolysate by fed-batch fermentation using Klebsiella oxytoca Process Biochem 2010 45 613 616 10.1016/j.procbio.2009.12.009

[B6] Wang AL Wang Y Jiang TY Li LX Ma CQ Xu P Production of 2,3-butanediol from corncob molasses, a waste by-product in xylitol production Appl Microbiol Biotechnol 2010 87 965 970 10.1007/s00253-010-2557-8 20376634

[B7] Ji XJ Huang H Du J Zhu JG Ren LJ Li S Nie ZK Development of an industrial medium for economical 2,3-butanediol production through co-fermentation of glucose and xylose by Klebsiella oxytoca Bioresour Technol 2009 100 5214 5218 10.1016/j.biortech.2009.05.036 19527928

[B8] Anvari M Pahlavanzadeh H Vasheghani-Farahani E Khayati G In situ recovery of 2,3-butanediol from fermentation by liquid–liquid extraction J Ind Microbiol Biotechnol 2009 36 313 317 10.1007/s10295-008-0501-z 19037672

[B9] Shao P Kumar A Recovery of 2,3-butanediol from water by a solvent extraction and pervaporation separation scheme J Membr Sci 2009 329 160 168 10.1016/j.memsci.2008.12.033

[B10] Li ZG Teng H Xiu ZL Aqueous two-phase extraction of 2,3-butanediol from fermentation broths using an ethanol/ammonium sulfate system Process Biochem 2010 45 731 737 10.1016/j.procbio.2010.01.011

[B11] Shin SH Kim S Kim JY Lee S Um Y Oh MK Kim YR Lee J Yang KS Complete genome sequence of Klebsiella oxytoca KCTC 1686, used in production of 2,3-butanediol J Bacteriol 2012 194 2371 2372 10.1128/JB.00026-12 22493189 PMC3347058

[B12] Celińska E Grajek W Biotechnological production of 2,3-butanediol – current state and prospects Biotechnol Adv 2009 27 715 725 10.1016/j.biotechadv.2009.05.002 19442714

[B13] Cripps RE Eley K Leak DJ Rudd B Taylor M Todd M Boakes S Martin S Atkinson T Metabolic engineering of Geobacillus thermoglucosidasius for high yield ethanol production Metab Eng 2009 11 398 408 10.1016/j.ymben.2009.08.005 19703579

[B14] Wang Q Chen T Zhao X Chamu J Metabolic engineering of thermophilic Bacillus licheniformis for chiral pure D-2,3-butanediol production Biotechnol Bioeng 2012 109 1610 1621 10.1002/bit.24427 22231522

[B15] Qin J Zhao B Wang X Wang L Yu B Ma Y Ma C Tang H Sun J Xu P Non-sterilized fermentative production of polymer-grade L-lactic acid by a newly isolated thermophilic strain Bacillus sp. 2–6 PLoS One 2009 4 e4359 10.1371/journal.pone.0004359 19194504 PMC2632756

[B16] Nazina TN Tourova TP Poltaraus AB Novikova EV Grigoryan AA Ivanova AE Lysenko AM Petrunyaka VV Osipov GA Belyaev SS Ivanov MV Taxonomic study of aerobic thermophilic bacilli: descriptions of Geobacillus subterraneus gen. nov., sp. nov. and Geobacillus uzenensis sp. nov. from petroleum reservoirs and transfer of Bacillus stearothermophilus, Bacillus thermocatenulatus, Bacillus thermoleovorans, Bacillus kaustophilus, Bacillus thermodenitrificans to Geobacillus as the new combinations G. stearothermophilus, G. th Int J Syst Evol Microbiol 2001 51 433 446 11321089 10.1099/00207713-51-2-433

[B17] Shu CK Lawrence BM Formation of 2-(1-hydroxyalkyl)-3-oxazolines from the reaction of acyloins and ammonia precursors under mild conditions J Agr Food Chem 1995 43 2922 2924 10.1021/jf00059a028

[B18] Xiao ZJ Xie NZ Liu PH Hua DL Xu P Tetramethylpyrazine production from glucose by a newly isolated Bacillus mutant Appl Microbiol Biotechnol 2006 73 512 518 10.1007/s00253-006-0491-6 16802153

[B19] Larroche C Besson I Gros JB High pyrazine production by Bacillus subtilis in solid substrate fermentation on ground soybeans Process Biochem 1999 34 667 674 10.1016/S0032-9592(98)00141-1

[B20] Xiao Z Ma C Xu P Lu JR Acetoin catabolism and acetylbutanediol formation by Bacillus pumilus in a chemically defined medium PLoS One 2009 4 e5627 10.1371/journal.pone.0005627 19461961 PMC2680964

[B21] Wu KJ Saratale GD Lo YC Chen WM Tseng ZJ Chang MC Tsai BC Su A Chang JS Simultaneous production of 2,3-butanediol, ethanol and hydrogen with a Klebsiella sp. strain isolated from sewage sludge Bioresour Technol 2008 99 7966 7970 10.1016/j.biortech.2008.03.062 18479913

[B22] Li ZJ Jian J Wei XX Shen XW Chen GQ Microbial production of meso-2,3-butanediol by metabolically engineered Escherichia coli under low oxygen condition Appl Microbiol Biotechnol 2010 87 2001 2009 10.1007/s00253-010-2676-2 20499229

[B23] Nielsen DR Yoon SH Yuan CJ Prather KLJ Metabolic engineering of acetoin and meso-2,3-butanediol biosynthesis in E. coli Biotechnol J 2010 5 274 284 10.1002/biot.200900279 20213636

[B24] Xiao ZJ Liu PH Qin JY Xu P Statistical optimization of medium components for enhanced acetoin production from molasses and soybean meal hydrolysate Appl Microbiol Biotechnol 2007 74 61 68 10.1007/s00253-006-0646-5 17043817

[B25] Alam S Capit F Weigand WA Hong J Kinetics of 2,3-butanediol fermentation by Bacillus amyloliquefaciens: effect of initial substrate concentration and aeration J Chem Technol Biotechnol 1990 47 71 84

[B26] Ma C Wang A Qin J Li L Ai X Jiang T Tang H Xu P Enhanced 2,3-butanediol production by Klebsiella pneumoniae SDM Appl Microbiol Biotechnol 2009 82 49 57 10.1007/s00253-008-1732-7 18949476

[B27] Zeng AP Biebl H Deckwer WD Production of 2,3-butanediol in a membrane bioreactor with cell recycle Appl Microbiol Biotechnol 1991 34 463 468

[B28] Zhang L Yang Y Sun J Shen Y Wei D Zhu J Chu J Microbial production of 2,3-butanediol by a mutagenized strain of Serratia marcescens H30 Bioresour Technol 2010 101 1961 1967 10.1016/j.biortech.2009.10.052 19932023

[B29] Gao J Xu H Li QJ Feng XH Li S Optimization of medium for one-step fermentation of inulin extract from Jerusalem artichoke tubers using Paenibacillus polymyxa ZJ-9 to produce R, R-2,3-butanediol Bioresour Technol 2010 101 7087 7093 20452206 10.1016/j.biortech.2010.03.143

[B30] Yu B Sun J Bommareddy RR Song L Zeng AP Novel (2R,3R)-2,3-butanediol dehydrogenase from potential industrial strain Paenibacillus polymyxa ATCC 12321 Appl Environ Microbiol 2011 77 4230 4233 10.1128/AEM.02998-10 21531839 PMC3131630

[B31] Garg SK Jain A Fermentative production of 2,3-butanediol: a review Bioresour Technol 1995 51 103 109 10.1016/0960-8524(94)00136-O

[B32] Johansen L Bryn K Stormer FC Physiological and biochemical role of the butanediol pathway in Aerobacter (Enterobacter) aerogenes J Bacteriol 1975 123 1124 1130 239921 10.1128/jb.123.3.1124-1130.1975 PMC235836

[B33] Magee RJ Kosaric N The microbial production of 2,3-butanediol Adv Appl Microbial 1987 32 89 161

[B34] Rodríguez H Fraga R Phosphate solubilizing bacteria and their role in plant growth promotion Biotechnol Adv 1999 17 319 339 10.1016/S0734-9750(99)00014-2 14538133

[B35] Eshkol N Sendovski M Bahalul M Katz-Ezov T Kashi Y Fishman A Production of 2-phenylethanol from L-phenylalanine by a stress tolerant Saccharomyces cerevisiae strain J Appl Microbiol 2009 106 534 542 10.1111/j.1365-2672.2008.04023.x 19200319

[B36] Chen TC Levin RE Taxonomic significance of phenethyl alcohol production by Achromobacter isolates from fishery sources Appl Microbiol 1974 28 681 687 4422523 10.1128/am.28.4.681-687.1974 PMC186797

[B37] Godtfredsen SE Rasmussen AM Ottesen M Rafn P Peitersen N Occurrence of α-acetolactate decarboxylases among lactic acid bacteria and their utilization for maturation of beer Appl Microbiol Biotechnol 1984 20 23 28

[B38] Pedersen S Lange NK Nissen AM Ann NY Novel industrial enzyme applications Acad Sci 1995 750 376 390 10.1111/j.1749-6632.1995.tb19982.x

[B39] Payne KA Hough DW Danson MJ Discovery of a putative acetoin dehydrogenase complex in the hyperthermophilic archaeon Sulfolobus solfataricus FEBS Lett 2010 584 1231 1234 10.1016/j.febslet.2010.02.037 20171216

[B40] Altschul SF Gish W Miller W Myers EW Lipman DJ Basic local alignment search tool J Mol Biol 1990 215 403 410 2231712 10.1016/S0022-2836(05)80360-2

[B41] Tamura K Peterson D Peterson N Stecher G Nei M Kumar S MEGA5: molecular evolutionary genetics analysis using maximum likelihood, evolutionary distance, and maximum parsimony methods Mol Biol Evol 2011 28 2731 2739 10.1093/molbev/msr121 21546353 PMC3203626

[B42] Xiao Z Huo F Huang Y Zhu X Lu JR A novel 2,3-xylenol-utilizing Pseudomonas isolate capable of degrading multiple phenolic compounds Bioresour Technol 2012 104 59 64 22074902 10.1016/j.biortech.2011.10.028

[B43] Stormer FC 2,3-Butanediol biosynthetic system in Aerobacter aerogenes Methods Enzymol 1975 41 518 533 236481 10.1016/s0076-6879(75)41108-9

[B44] Steinbüchel A Fründ C Jendrossek D Schlegel HG Isolation of mutants of Alcaligenes eutrophus unable to derepress the fermentative alcohol dehydrogenase Arch Microbiol 1987 148 178 186 10.1007/BF00414809

